# Supplementary figures and images for: Use of soil spectral reflectance to estimate texture and fertility affected by land management practices in Ethiopian tropical highland
Source: PLoS One. 2022 Jul 21;17(7):e0270629. doi: 10.1371/journal.pone.0270629 (PMC9302783; doi:10.1371/journal.pone.0270629)

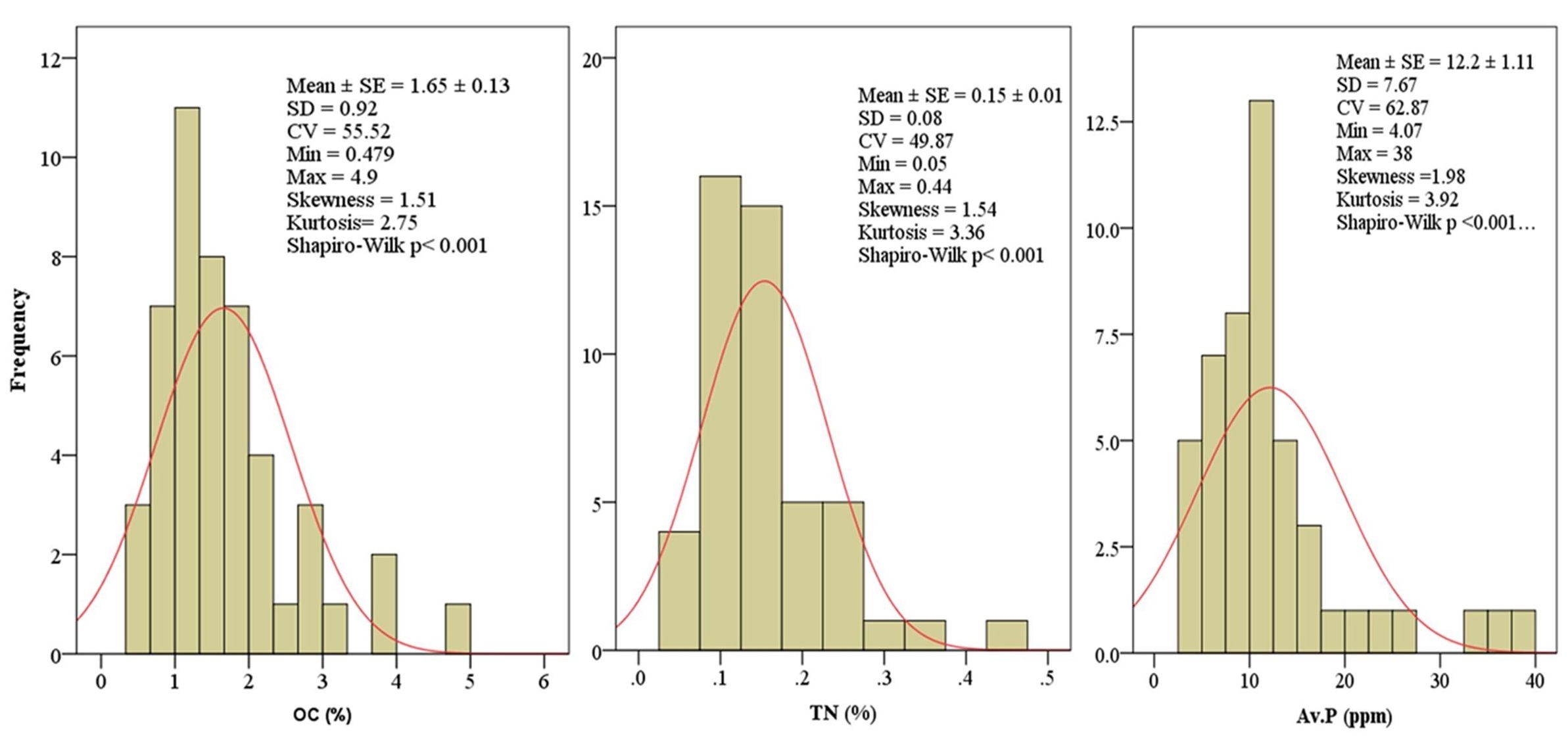

Supplement: S1 Fig — SE, standard error; SD, standard deviation; CV = coefficient of variation; min, minimum; max, maximum. (JPG) [file pone.0270629.s001.jpg]

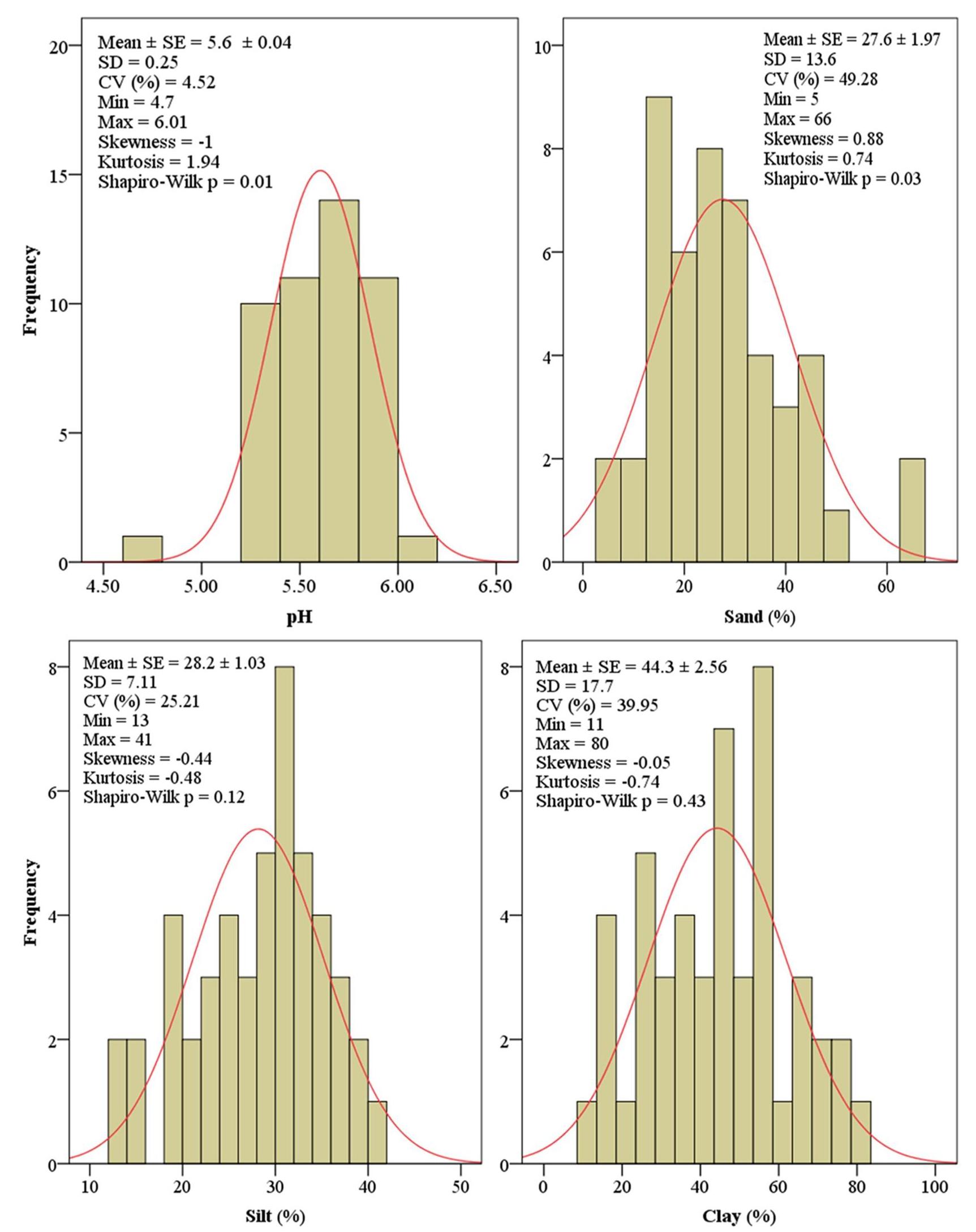

Supplement: S2 Fig — SE, standard error; SD, standard deviation; CV = coefficient of variation; min, minimum; max, maximum. (JPG) [file pone.0270629.s002.jpg]

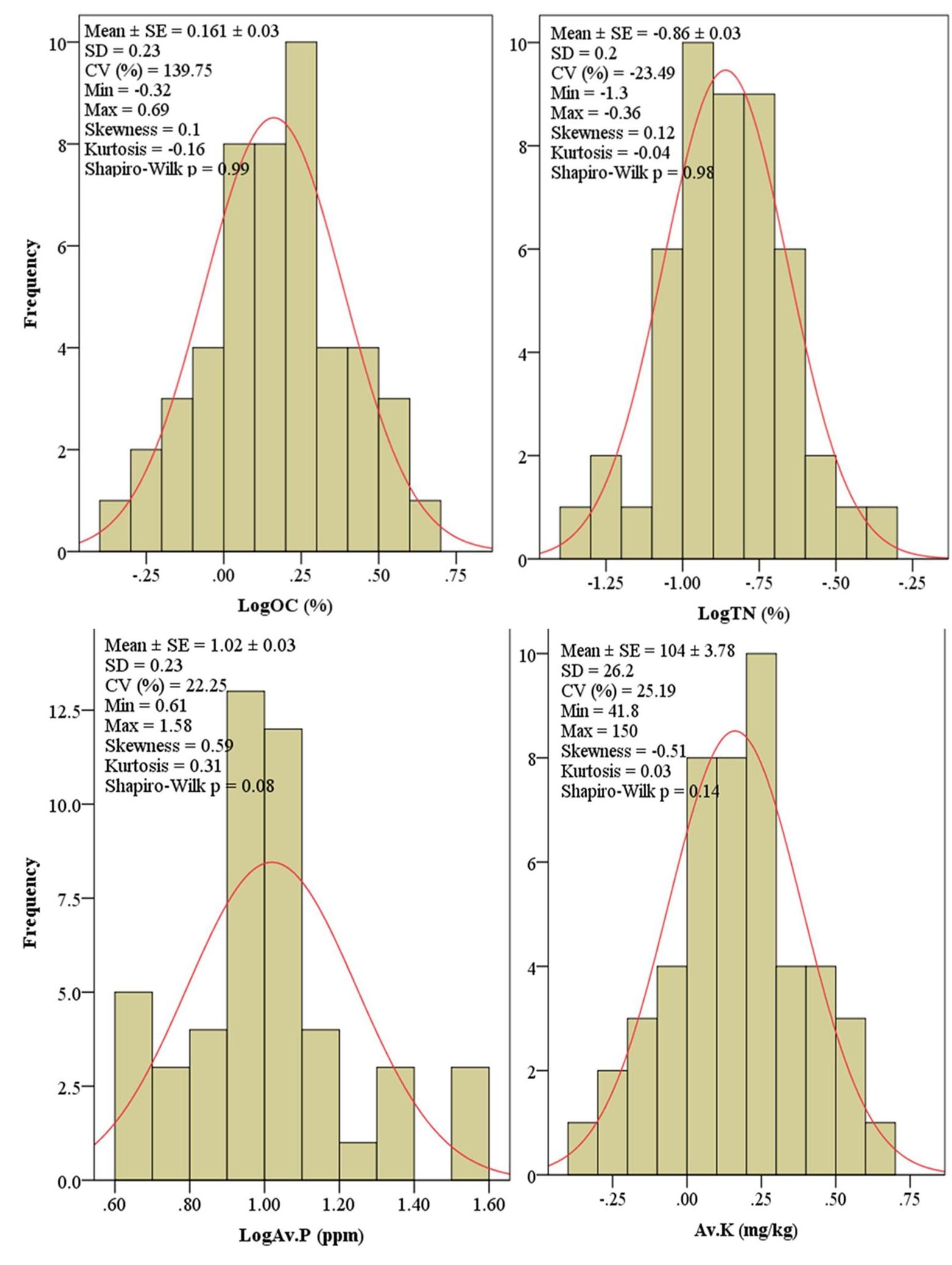

Supplement: S3 Fig — Log, logarithmically transformed; OC, organic carbon; TN, total nitrogen; av. K, available phosphorus; available potassium, min, minimum and max, maximum. (JPG) [file pone.0270629.s003.jpg]

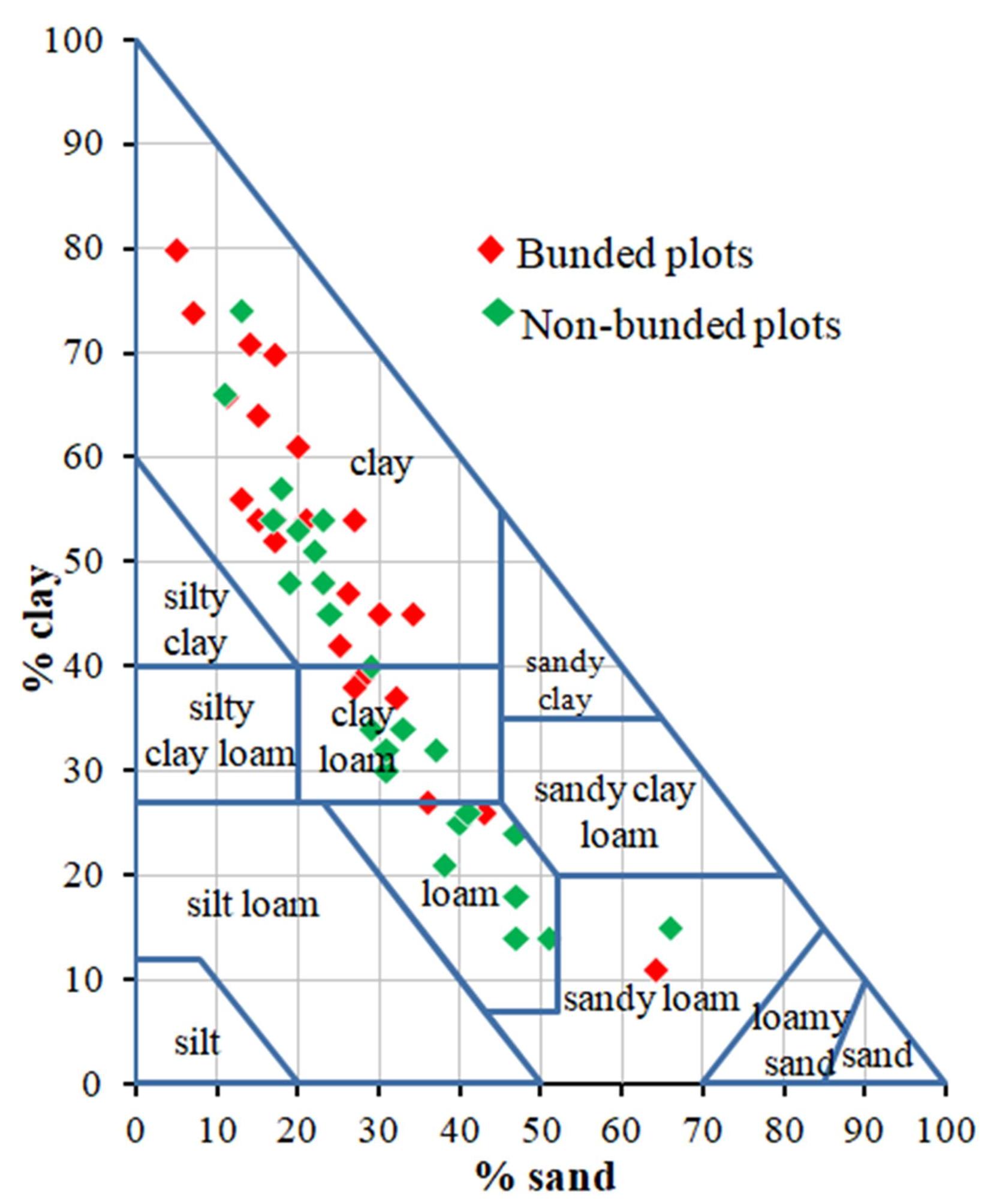

Supplement: S4 Fig — (JPG) [file pone.0270629.s004.jpg]
